# Development of a Physical Therapy-Based Exercise Program for Adults with Down Syndrome

**DOI:** 10.3390/ijerph20043667

**Published:** 2023-02-18

**Authors:** Sarah Mann, Jennifer Spiric, Cailin Mitchell, Thessa Irena Maria Hilgenkamp

**Affiliations:** 1 Mann Method PT and Fitness, Arvada, CO 80005, USA; 2 Department of Physical Therapy, University of Nevada, Las Vegas, NV 89154, USA

**Keywords:** Down syndrome, physical therapy, exercise, musculoskeletal, motor competence

## Abstract

In adults with Down syndrome, the combination of low physical activity and fitness levels and the high prevalence of musculoskeletal co-morbidities stresses the need for specialized exercise programs. The goal of this research study was to develop a specialized exercise program for individuals with Down syndrome using the physical therapy approach of a systems review as the foundation. We first conducted an overview of the literature on co-morbidities in adults with Down syndrome using the systems review method to categorize these findings. We extracted recommendations for content and delivery of an exercise program based on the literature review, and finally composed a specialized exercise program for individuals with Down syndrome adhering to these recommendations.

## 1. Introduction

Down syndrome (Ds) is an autosomal disorder caused by the presence of an extra chromosome 21, also known as trisomy 21 [1]. Statistics vary, but it is estimated that over 200,000 people in the United States today have Ds [2]. Ds causes a range of intellectual disabilities and developmental delays in motor skills. The lifespan of people with Ds has increased from an average of 12 years in the 1940s [3] to 60 years now for people with Ds in high-income countries [4]. Most adults with Ds lead a sedentary lifestyle; previous research reported that less than 10% of adults with Ds achieve the recommended amount of physical activity [5,6]. Adults with Ds often experience cardiovascular issues and generally higher rates of obesity than their peers [7], and low aerobic capacity [8]. In addition to lower cardiovascular function, individuals with Ds exhibit lower strength compared with other sedentary peers [9,10]. Both poor cardiovascular fitness and lower muscle strength contribute to increased fatigue and difficulty with activities of daily living, such as rising from a chair, ascending and descending stairs, and walking [11]. Individuals with Ds also report barriers to physical activity that involve ‘physical and medical factors’, including obesity and cardiovascular concerns [12].

**Figure 1:**
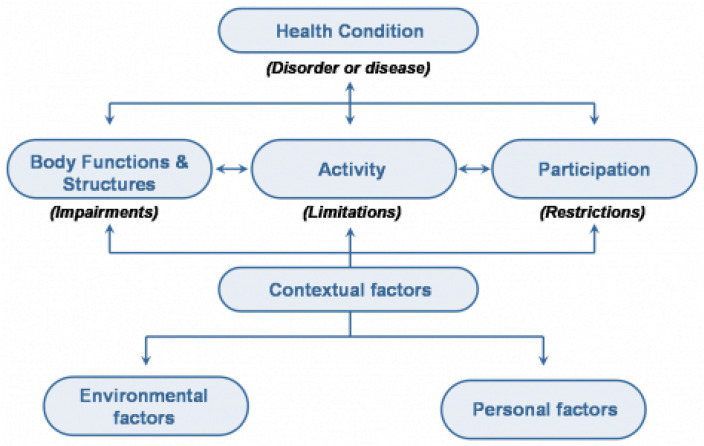
ICF model [20]. World Health Organization (2001). License: CC BY-NC-SA 3.0 IGO.

Previous studies have addressed these issues by implementing and evaluating exercise interventions for individuals with Ds. Recent systematic reviews by Li et al., Paul et al., and Palomba et al. support that exercise programs can improve strength, cardiometabolic risk profile, and aerobic endurance in individuals with Ds [13,14,15]. Although physical therapists have an important role in supporting children with Ds (gross motor skills, mobility, endurance, strength), they are less frequently involved in exercise programming for adults with Ds, mostly due to difficulties with funding and access to specialized care [16,17]. Due to increased life expectancy and specific genetic physical features, combined with a low active lifestyle, adults with Ds need specialized assistance with, or rehabilitation of, functional activities and skilled pain management [17,18]. The expertise of a physical therapist can greatly contribute to increased activities, participation, and rehabilitation in adults with Ds by applying a systems-based physical therapy (PT) approach. In this specialized approach, functional impairments are categorized in the different ‘systems’ of the body, such as the cardiovascular system, the musculoskeletal system, the neurological system, etc., hence ‘systems-based’ [19]. This approach is rooted in the World Health Organization’s International Classification of Functioning, Disability and Health (ICF) model (Figure 1). The ICF model is a framework that conceptualizes the relationships between body structure and function, the ability to perform activities, and (social and societal) participation [20]. Impairments (at the body structure and function level) have been shown to impact activities and participation [20]. Therefore, interventions targeting physical activity and exercise could increase their impact by including strategies to address impairments and activity limitations and targeting participation as the main goal [20].

The ICF model includes contextual factors (consisting of environmental and personal factors) that impact impairments, activities, and participation. These contextual factors are important determinants of physical activity behavior and have been thoroughly described in reviews by others, such as Mahy et al. (2010) and Bartlo et al. (2011) [12,21]. This paper aims to contribute to our understanding of how to best support physical activity and fitness in individuals with Ds by taking the specialized expertise of physical therapists as the starting point to accommodate and address the specific cardiovascular, musculoskeletal, and neuromuscular impairments that are associated with Ds. In this paper we will focus on impairments in body structure and function that impact the ability to perform physical activity, such as cardiovascular function ligamentous laxity, joint range of motion and alignment, hypotonia, balance, and an increased risk for osteoporosis with age [22,23,24]. A thorough physical therapy-based ‘systems review’ for adults with Ds offers a comprehensive starting point for exercise program design, yet has not been promoted in previous exercise interventions for individuals with Ds. The systems-based PT approach aims to improve impairments at the ‘body structure and function level’ (Figure 1), thus making the ‘Activities’ of the exercise program safer, more efficient, and successful, with the overall goal of improving ‘Participation’. To the best of our knowledge, there is currently no evidence-based exercise program available for adults with Ds that addresses the underlying impairments of adults with Ds in order to improve activities and, ultimately, participation. We therefore aimed to apply this systems-based PT approach, based on the ICF model, to design an evidence-based exercise program for adults with Ds that addresses their impairments while improving activities and participation. This exercise program will not just be usable for physical therapists but will be available for use to all rehabilitation professionals and exercise specialists working with individuals with Ds.

## 2. Materials and Methods

Starting with the ‘Body Structure and Function’ of the ICF model, a literature search was performed to support a physical therapy-based ‘systems review’, with an overview of ‘Impairments’ that impact ‘Activities’ (see Figure 1) for individuals with Ds. This ‘systems review’ is the framework that physical therapists use to examine their patients [19,25]. It organizes impairments in cardiovascular/pulmonary, integumentary, musculoskeletal, and neuromuscular systems, as well as the patient’s cognitive, language, and learning abilities. We searched the following databases between December 2021 and December 2022: Pubmed, Embase, and CINAHL. We used wide search terms referring to these systems combined with ‘Down syndrome’ or ‘trisomy 21’. We further used the review papers that resulted from this search to find original papers and used reference lists of all papers to find other applicable work; however, the literature was scarce on many of the systems and/or impairment for adults with Ds. In this paper we have narrowed our focus to impairments in individuals with Ds directly impacting physical activities, but for possible interventions we had to expand our search to other populations for some impairments. For a more comprehensive review of co-morbidities in Ds, we refer to two recent reviews of the literature by Capone et al. [7] or earlier overview papers, such as Bittles et al. [26]. We translated these findings into recommendations for both content and delivery of an exercise program for individuals with Ds, organized by the same categories of the systems review described above. Recommendations for the content of the program will be discussed according to the FITT criteria formulated by the American College of Sports Medicine: Frequency, Intensity, Time, and Type [27]. Finally, a detailed exercise program based on these recommendations is presented.

## 3. Results

### 3.1. Systems Review

Table 1 presents a comprehensive overview of ‘Impairments’ in body structure and function that limit ‘Activities’ for individuals with Ds. The second column provides prevalence rates where available, while brief examples of how those impairments present themselves are described under ‘Implications’, and current Potential Interventions are included in the last column.

With regard to the cardiovascular/pulmonary system, individuals with Ds often have impaired cardiovascular function, decreased endurance [11], lower maximal heart rates [28], lower blood pressure [29], and impairments of the heart and valve function itself. For exercise prescription, it is important to note that lower blood pressure can impact the feasibility of certain exercises, and including extra time for transitions can prevent lightheadedness [30]. Individuals with Ds often have significant involvement of the musculoskeletal system, including ligamentous laxity [22] and low muscle tone [23], leading to a higher risk of pes planus, scoliosis, hip disorders, and patellar instability if untreated [7,22,23,31,32]. Strengthening of core muscles and joint stabilizers are frequently recommended strategies to counteract the effects of ligamentous laxity and low muscle tone [33,34,35,36,37]. Atlanto-axial instability is a contraindication for exercise, although its prevalence is low [7,38]. Osteoporosis seems to be more prevalent in individuals with Ds [9], as does inflammatory arthritis in children with Ds [22,24]. Obesity can further negatively impact joint alignment, gait mechanics, cardiovascular endurance, and joint range of motion. Neuromuscular system involvement in individuals with Ds contributes to delayed gross motor skill development [39]. Gait and balance can be decreased, with a wide base of support and difficulty with daily activities [40,41]. Balance can also be impacted by the high prevalence of visual, hearing, vestibular, and sensory integration impairments [40,41,42,43,44,45,46,47]. Cognitive, language, and learning abilities in individuals with Down syndrome include intellectual disability, difficulties with expressive speech, and delayed processing speed [48,49,50]. Individuals with Ds are often excellent visual learners who respond best to positive reinforcement strategies [48,49,50].

**Table 1:** Systems review with overview of impairments, prevalence, implications, and possible interventions.

Impairments	Prevalence in Population with Ds	Implications	Possible Interventions
Cardiovascular/pulmonary			
Impaired Heart Rate Regulation [28]	Unknown	Lower than expected heart rates at all intensities [28].	Ds-specific formula to predict maximal heart rate: 179 − (0.56 × age) [28]
Cardiovascular Dysfunction [11]	Unknown	Decreased endurance, ability to perform daily activities [11].	3–7 days/week at 40–80% of VO2R or HRR, 30–60 total min/day, preferably walking; or running, swimming, stationary cycling [49] When sedentary, ‘start low and go slow’ [51]
Impaired Blood Pressure Regulation [29]	Unknown	Low blood pressure [29], lightheadedness, orthostatic hypotension [52].	If symptoms are present: changes in diet and fluid intake, awareness of body position changes, or medication [30]
Impaired Heart Valve Structure [7]	Mitral valve disease (prolapse or regurgitation) 36%, tricuspid disease (insufficiency or regurgitation) 10%, aortic disease (insufficiency or regurgitation) 8% [7]	Valve dysfunction [7], shortness of breath, difficulty catching your breath, fatigue, weakness, or inability to maintain regular activity level, lower cardiovascular capacity [53].	Surgery; post-surgical rehabilitation with cardiac therapy [53]. Possible need for exercise intensity modifications.
Impaired Pulmonary Pressure Regulation [54]	Associated with congenital heart disease or upper airway obstruction [54]	Pulmonary hypertension [54], fatigue, decreased energy and participation [55]. Possible supplemental oxygen needs.	Surgery for heart defects and treatment of airway obstruction, vasodilator therapies, supplemental oxygen [54]. Possible need for exercise intensity and duration modification.
Musculoskeletal			
Impaired Metabolism [7,56]	Overweight: 38% Obese: 34% [7]	Obesity, impacted gait [56], decreased energy, decreased motivation, decreased physical activity [57].	Multifactorial interventions including physical activity, diet and behavioral change [56]
Ligamentous Laxity [22,23]	100% [23]	Increased range of motion at all joints, plays a role in flat feet, hip disorders, patellar instability, atlanto-axial instability, poor grip strength, difficulty with dexterity and fine motor activities, atypical gait [40,41].	Strength exercises to strengthen the muscles surrounding the joints for added support [33,36]. To improve gait: treadmill interventions, orthoses [58]
Pes Planus [22,23,31]	60–76% [23]	Increased risk for hallux valgus, bunions, great toe abduction, atypical gait, decreased gait speed, decreased step length, fatigue with walking/standing, knee pain, decreased motivation to move [31].	Orthotic foot support, insoles, inserts and proper shoes [23,59]
Hypotonia (low tone) [23]	At least 80% [23]	Resting muscle tone, commonly confused with inability to build strength.	Support for PT and OT interventions focused on improving strength and motor planning [36]
Scoliosis [22]	4.8% [22]	Decreased abdominal strength and endurance, decreased trunk strength and endurance, decreased scapular strength and endurance, decreased glenohumeral joint range of motion, compensation patterns for upper extremity movement, leg length discrepancy, atypical gait pattern, radicular pain, pain in neck, back, hip, knee or leg [35].	Remediate: Core strengthening, trunk musculature strengthening, scapular strengthening. Compensate: foot support, shoe lift, bracing [35].
Hip Disorders [7,60]	Between 5 and 20% [7], 28% [60]	Dislocation, dysplasia, and impingement [7].	Strengthen dynamic stabilizers, or surgical treatment [34,61], total hip replacement [7]
Patellar Instability/Dislocation [32]	4–8% [32]	Usually associated with ligamentous laxity. Knee pain, decreased gait endurance, decreased gait speed, fear of participating in dynamic activities.	Functional/asymptomatic: conservative rehabilitation [37] Severe/affecting functioning: surgical intervention [37].
Atlanto-Axial Instability [7]	2–20% [7]	Avoid activities that increase risk for atlanto-axial dislocation [38].	Surgery/Avoid activities that increase risk for atlanto-axial dislocation [38]
Spondylosis or Degenerative Change of the Cervical Spine	33–64% (age-dependent) [7]	Possible pain, possible decreased muscle strength.	Surgical decompression-stabilization, specific exercises to maximize function and decrease pain; Modifications to exercise positions and movement ranges to protect cervical spine and nerves.
Decreased Muscle Strength [8]	Unknown	Decreased ability to perform daily activities [8].	Progressive strength exercise training program targeting major muscle groups following ACSM guidelines [49]
Osteoporosis [7,9]	Increased risk compared to peers in general population [7]	Increased risk of fracture [62].	Multifactorial interventions focused on physical activity, sunlight exposure and vitamin D [7] Dynamic (active) weight bearing [62]
Arthritis [22,24]	7% inflammatory arthritis in children with Ds [22]	Stiffness, pain, avoidance of physical activities [63].	Medication [63], moderate exercise
Neuromuscular			
Impaired Balance [47]	Unknown	Impaired static balance, problems with altered somatosensory input [47], atypical gait [40,41].	Various exercise programs to improve balance in anteroposterior and mediolateral directions, treadmill walking, core stabilization, visual-vestibular integration [47,64] Core stability exercises, isokinetic strengthening, and treadmill training [65,66,67,68]
Visual Impairment [43,44]	78% in adults with Ds [69] Increased incidence of nystagmus and strabismus	Issues with focus [43], depth perception, color discrimination, and reduced sensitivity [44].	Appropriate eye wear and/or accommodations
Hearing/Vestibular Impairments [42,45,46]	Hearing impairment up to 73% [45]	Documented differences in inner ear anatomy/shape may impact vestibular function [42,46].	Appropriate hearing aids and/or accommodations. For vestibular impairments: visual-vestibular exercises [70]
Impaired Proprioception [47]	Unknown	Children with Ds have difficulty interpreting somatosensory input to achieve postural control for maintaining balance [47]. Decreased feedback from proprioceptive sensors in joints with ligamentous laxity [71].	Balance training, visual-vestibular exercises [64,70]
Seizures [72]	1–13% [72]	Can develop in infancy but also in the third decade of lifespan [72].	Medication, safety measures [72]
Cognitive, language, and learning abilities			
Cognitive Impairment [48,49,50]	Majority of individuals with Ds. Varied degree of cognitive impairment [48]	Slower processing time [48]. Varied degree of cognitive impairment [48]. Difficulty with expressive speech language, speech intelligibility [48]. Potentially reduced and delayed pain responses, not insensitive to pain, but expression of pain is often is delayed and less precise [48]. Preference for sameness and routine [48]. Preference for routines and ‘grooves’ [48]. Difficulty with generalization Excellent visual learners [48].	Motivated by positive social encouragement [48]. Effective strategies include positive reinforcement [50]. Use simple, one-step instructions [49]. Appropriate familiarization and practice needed [49]

### 3.2. Results for Program Content

ACSM guidelines recommend that individuals with Ds receive a physical evaluation prior to engaging in any kind of exercise testing or subsequent training, with specific attention to joint instability (including atlanto-axial instability [38] and cardiovascular impairments [49]).

#### 3.2.1. Cardiopulmonary

##### Frequency, Intensity, and Time of Exercise

The decreased endurance in individuals with Ds warrants the inclusion of aerobic exercise in an exercise program [11]. The intensity needs to be based on the Ds-specific formulas for prediction of maximal heart rate, as this is lower than in the general population [28] Following the guidelines in the section on Intellectual Disability and Down Syndrome by the American College of Sports Medicine [49], adults with Ds are recommended to engage in moderately intense to intense activities (40–80% of VO2max) at least 3 days per week for at least 30 min/day. It is important to start with lower-intensity activities and gradually increase frequency and duration to accommodate for the decreased endurance. The Ds-specific formula for prediction of maximal heart rate is 179 − (0.56 × age) [28], which results in different heart rates to guide relative intensity of exercise (% of maximal heart rate). Monitoring heart rate throughout the exercise session with a heart rate monitor or mobile technology helps to avoid relying on estimates of intensity by an observer, as this may lead to underestimation of actual effort. As for any individual starting aerobic exercise, the American College of Sports Medicine recommends performing a pre-exercise health screening by a health care professional [27].

##### Types of Exercises

Exercises without the use of large fitness equipment provide a viable, easily accessible option to engage in cardiopulmonary exercise. Walking and dancing, either guided or independent, are widely used types of exercise that can meet the requirements with regard to intensity. Sequencing strength exercises and large muscle group movements back to back also elicit a cardiovascular effect. Individual strength exercises are discussed below, but an effective cardiovascular sequence is squats, squat and reach, squat jumps, side-to-side jumps, adduction/abduction jumps, and standing marches—all exercises in a row with minimal rest between each exercise.

Exercises to improve cardiopulmonary fitness can also incorporate cardiovascular equipment, such as rowing machines, ellipticals, recumbent and upright bicycles, and treadmills [49]. The use of equipment can offer an additional focus on improving foot position, gait mechanics, reciprocal movement patterns, weight shift, and balance while working on cardiovascular improvements [65,66,67,68]. Individual preferences, body type, balance, and sequencing ability should be taken into consideration when selecting the most suitable equipment. If available, variation between machines can be effective (for example: 5 min intervals on a different machine each interval—5 min bike, 5 min elliptical, 5 min treadmill, 5 min rowing machine), as well as including limited breaks through an exercise routine.

#### 3.2.2. Musculoskeletal

##### Frequency, Intensity, and Time of Exercise

Due to the presence of ligamentous laxity [21,22,31], and the lower muscle strength in adults with Ds [8], strength exercises are highly recommended for adults with Ds, with a specific emphasis on strengthening lower extremity, hip, core, trunk, upper extremity, and scapular musculature, using dynamic exercise. ACSM guidelines for strengthening exercise for individuals with intellectual disabilities and Ds follow the principles of programming for a novice with a focus on major muscle groups [49]. Each major muscle group should be trained 2–3 times per week, and the intensity should start at 60–70% of 1RM (one repetition maximum) for novice participants (10–12 repetitions, 2–3 sets), gradually increasing to 70–80% of 1RM (10–12 repetitions, 2–3 sets) [49]. No specific duration of training is recommended.

##### Types of Exercises

Due to the ongoing development and refinement of gross motor skills, exercises involving functional whole-body movements are preferred over smaller movements targeting isolated muscle groups. Although ACSM guidelines prefer machines for resistance exercise, exercises without equipment are very suitable for achieving the goals of strengthening and stabilizing if supervised by a trained professional. Exercises for adults with Ds need to be age-appropriate and are most effective if taught with simple and concise cueing [48,49]. Recommended exercises are categorized as Foundational Exercises or Hip Strengthening Exercises, both including core stabilization exercises.

Foundational Exercises:

Given the predisposition for ligamentous laxity, hypotonia, pes planus (flat feet), genu valgum, genu recurvatum, decreased hip stability, decreased abdominal activation, and scoliosis (see Table 1), global strengthening exercises are critical for exercise prescriptions for individuals with Ds. Foundational movements such as squats, push-ups, planks, and gluteal bridges foster activation and strength of the abdominal, gluteal, hip, trunk and upper extremity musculature, hip stability, and improved neuromuscular sequencing.

Squats target the lower extremity musculature and improve concentric and eccentric muscle activation of the gluteal muscles, hamstrings, and quadriceps. Squats foster a dynamic gastrocnemius/soleus stretch with heel contact and anterior tibial translation with knee flexion. Squats also improve trunk extension and core stabilizers with abdominal activation and bracing when performing the movement correctly. Improvements in squat strength and sequencing can directly improve sit-to-stand and stand-to-sit transfer patterns.

Push-ups are a multi-joint exercise that strengthens scapular stabilizers and upper extremity musculature. Push-ups also foster abdominal and gluteal coactivation, lumbar stabilization, hip stabilization, and neutral hip positioning for a population at higher risk for scoliosis and hip instability.

Planks are a core strengthening activity that increases abdominal and gluteal strength, abdominal and gluteal coactivation around the hip and spine, upper extremity proprioceptive input, and strengthens the scapular and upper extremity muscles. With a high likelihood of a flat-footed position with often associated plantarflexion and external rotation, the plank on toes also allows for individuals with Ds with a tight gastrocnemius/soleus complex to achieve a stretch through these muscle groups in a position of neutral dorsiflexion.

Gluteal bridges are a gentle and effective way to achieve abdominal and gluteal activation for core and hip musculature strengthening, and neutral spinal positioning with the hook lying position on the floor. 

Hip Strengthening Exercises:

With increased ligamentous laxity, gait asymmetry, and gait inefficiencies, individuals with Ds have a high incidence of hip dysfunction, including hip dysplasia, hip dislocation, hip impingement, and general hip impairment [7,73]. Hip-specific strengthening activities target gluteal and lateral hip musculature to improve hip strength and stability, weight-shift and functional mobility for balance, and reductions in pain, as indicated in studies on the general population for hip stabilization [34].

Marching recruits hip flexors, core musculature, and lateral hip stabilizers in a single-leg stance. It can be progressed from sitting to standing, performed as an ipsilateral (same side for consecutive reps) or alternating activity, and completed with and without hand support to meet the needs of individuals relative to their balance and processing.

Hip abduction is a lateral hip musculature strengthener, concentrically with the lifting leg and isometrically with the stance leg. Hip abduction is effective on its own and as part of the visual-vestibular lateral tilts with targeted strengthening and stabilizing through the gluteus maximus and medius for hip strength and single-leg stability.

Tall kneeling and split stance movements target hip strengthening and functional transfers. Individuals with Ds often develop transfer patterns moving from stand to sit through full hip and knee flexion with increased hip external rotation, putting increased stress on the hip joint. Tall kneeling strengthens hip and knee adductors, gluteal muscles, and core stabilizers and works postural righting patterns with upright postural control. Tall kneeling can be paired with upper extremity movements to increase complexity and difficulty. Moving through split stance (lunge or surrender) strengthens and stabilizes the hip musculature and provides a sequence for transfers to and from the floor, decreasing the demand on the hip joint when compared to full hip flexion and external rotation.

Quadruped is a widely used exercise for core, trunk, and hip stabilization [74]. It is prescribed for low back pain and hip stabilization in the general population and provides closed-chain strengthening options for the upper extremities, scapular musculature, trunk musculature, core musculature, and hip musculature [74]. It can be progressed with variation in closed and open chain positions and provides an additional benefit of increased upper extremity proprioceptive input.

#### 3.2.3. Neuromuscular

##### Frequency, Intensity, and Time of Exercise

Balance exercises are recommended [49], as adults with Ds experience balance deficits due to visual impairment, hearing impairment, vestibular nerve (CN VIII) impairment, documented differences of inner ear anatomy, diminished vestibular ocular reflex, and decreased proprioception due to ligamentous laxity (see Table 1). Evidence shows that specific exercises improve balance, function, and independence for people with Ds [64].

Most individuals with Ds present with ligamentous laxity, hypotonia, and joint hypermobility. However, with the prevalence of a flat-footed posture, gait asymmetry, and scoliosis, many individuals with Ds develop postural and muscular asymmetry over time, as well as decreased muscle length in some muscle groups. Therefore, specific stretching is also an important part of an exercise program, and following ACSM guidelines is preferred to be included daily, but at least 2–3 times/week [49].

##### Types of Exercises

Visual-Vestibular exercises:

To improve balance systems and their integration, specific visual-vestibular exercises, such as lateral tilts, rotational passes or taps, anterior/posterior tilts, and over-under passes or taps, are recommended. These exercises improve gait stability, postural control, functional weight shifts, righting reactions, visual-vestibular coordination, dynamic single-leg standing balance, and integration of sensory information coming from the visual and vestibular systems during movement. When combined with stabilization patterns through the hips and shoulder girdles, postural changes and stability with dynamic single-leg stance are enhanced. When progressed in combination with a visual target or specific visual cues for eye focus, gaze stability is improved, and visual-ocular reflex integration occurs, resulting in greater balance and stability. Exercise equipment for visual-vestibular exercises utilizes distinct colors to support visual and tactile feedback for precision of movement and integration of multiple systems working together.

Over-under passes is an adaptation of the Catch a Falling Star exercise in the Astronaut training program [70]. It combines vestibular activation during cervical extension and cervical flexion movements with upper body reaching overhead and under legs and includes a visual target-finding component with head and body movements in the sagittal plane. It can be progressed from sitting to standing while holding a small object at the midline using both hands. It can be performed while sitting/standing with the individual’s back to the wall or while sitting/standing back-to-back with a partner.

Rotational Passes is an adaptation of the Robot Zapping exercise in the Astronaut training program [70]. It combines upper trunk rotation with hands at midline with a reciprocal lateral weight shift and includes a visual target-finding component with head and trunk rotation to achieve visual-vestibular coordination in the transverse (rotational) plane. It can be progressed from sitting to standing and performed with one hand reaching across the body or two hands holding a small object at midline. It can be performed while sitting/standing with the individual’s back to the wall or while sitting/standing back-to-back with a partner.

Rotational passes with diagonal bias is an adaptation of the Moonboot Dusting exercise from the Astronaut training program [70]. It is a progression on rotational passes and combines upper trunk rotation, cross midline reaching, and visual target-finding during a diagonal movement with the head and trunk through space to activate and coordinate multiple parts of the vestibular systems with unique visual coordination patterns. It can be progressed from sitting to standing and is performed with both hands holding a small object at the midline.

Lateral tilts are a functional exercise that encourages lateral translation of body weight from medial to lateral across the foot and improves postural control with side-to-side weight shifts from one foot to the other. As a standing exercise, it strengthens lateral hip stabilizers in dynamic single-leg stance. As a seated exercise, it facilitates upper trunk righting reactions, forward gaze, and postural control. It can be progressed from sitting to standing, performed with and without hand support, and enhanced with a visual challenge of locking gaze on a specific focal point.

Anterior/Posterior tilts are a functional exercise that encourages an individual to activate intrinsic foot muscles, control body weight translation across the foot between the heel and ball of foot, and improve postural control in the sagittal plane. As a standing exercise, it also encourages right/left dissociation and trailing limb posture and strengthens lateral hip stabilizers in a dynamic modified tandem stance. It is performed in a standing position, can be progressed with and without physical assistance at the waist or hand support, and is easily enhanced with a visual challenge of locking gaze on a specific focal point.

Stretches:

Stretching and range of motion exercises that emphasize postural control and postural symmetry, including hip flexor, lumbar extensor, hamstring, and gastrocnemius/soleus mobility are appropriate for individuals with Ds.

Chest openers activate scapular retractors, achieve thoracic extension, and incorporate a dynamic pectoral stretch. Due to their prevalence of scoliosis and decreased muscle tone, individuals with Ds often sit and stand in a position of increased thoracic flexion, cervical flexion, a downward gaze, and increased bilateral scapular protraction. Chest openers allow for a dynamic and rhythmic stretch of the tight anterior pectoral muscles without placing prolonged force or pressure against the hypermobile glenohumeral joint, while creating dynamic scapular retraction and thoracic extension.

Overhead reaches activate the scapular and glenohumeral musculature and thoracic extension. Overhead reaches allow for dynamic and rhythmic activation of the glenohumeral and scapular musculature paired with trunk extension with overhead reach and dynamic abdominal activation with overhead reach.

Supine single knee to chest allows for a supported hip flexor stretch of the iliopsoas, tensor fascia latae (TFL), and rectus femoris in a supported position. With their common position of pes planus, foot external rotation, hip external rotation, decreased abdominal activation, anterior pelvic tilt, and asymmetries through the gait cycle, individuals with Ds often present with tight hip flexors, hip external rotators, and lumbar extensors. The single knee to chest is a gentle stretch, performed lying on the person’s back, that achieves a stretch of the lumbar extensors on the ipsilateral side of the flexed hip and knee and a stretch of the contralateral hip flexors (on the extended lower extremity side).

Hurdler stretch allows for a supported stretch of the lumbar extensors, hamstrings, and gastrocnemius/soleus complex. It targets muscle tightness secondary to patterns of pes planus, foot external rotation, hamstring overfiring in standing, increased plantar flexion, scoliosis, postural preferences, and asymmetries throughout the gait cycle common in individuals with Ds. In the hurdler position, the individual moves into the position of lumbar flexion, hip flexion, knee extension, and ankle dorsiflexion, achieving a stretch through the targeted muscle groups.

Calf stretch, performed in supine with a non-elastic strap, provides a supported stretch to the gastrocnemius/soleus complex. Because of their pes planus, foot external rotation, increased plantarflexion, and gait asymmetries, teens and adults often present with notable calf hypertrophy and limitations in ankle dorsiflexion. Muscle tightness through the gastrocnemius/soleus complex can lead to foot pain, plantar fasciitis, and decreased walking endurance. The calf stretch, performed in supine, with the strap placed at the ball of the foot and both knees extended, provides a position of supported ankle dorsiflexion, stretching the plantar flexors, and requiring no additional standing balance for the activity.

### 3.3. Results for Program Delivery

Individuals with Ds have unique learning styles, learning strengths, and cognitive impairments that require special considerations to optimize program delivery to achieve a successful outcome (see Table 1). To achieve successful implementation of a PT-based exercise program for adults with Ds, it is critical to focus on setting up the participants for success, practicing specific movements in the program over time with specific verbal, tactile, and visual cues, progressing the program slowly to allow for adjustment time, and emphasizing consistent practice and a good routine. A consistent exercise order and concise cueing of the exercises can greatly support participant success and confidence [48]. Adults with Ds are great visual learners and do very well with visual charts and visual demonstrations [48]. Adults with Ds have a great adherence to routine, allowing for building experience and mastery of exercises [48]. Positive reinforcement is important throughout the program [50]. They benefit from supportive and knowledgeable supervision that incorporates these learning preferences [50]. Providing direct attention, using concise instructions, using consistent language, and providing appropriate processing time to process the request are effective strategies when working with adults with Ds [48].

### 3.4. Detailed Exercise Program

Based on the results for program content and delivery, an exercise program for adults with Ds was composed: the Mann Method PT Exercise Program (see the Supplemental Materials for detailed Exercise Prescription Chart, including pictures). These specific exercises require minimal equipment, can be performed independently, and can effectively improve walking patterns, strength, stability, balance, coordination, endurance, and participation in purposeful activity with increased independence. The Mann Method PT Exercise program is designed to be executed 3 times per week, about 1 h per session, and can be performed in various settings, such as a physical therapy clinic, a fitness facility, or at home, due to the consistent exercise order and the need for only basic exercise materials (exercise balls and a mat). While individuals can participate in the program independently, it is best to perform this program under the supervision of a licensed and trained physical therapist who is skilled in correcting form, optimizing movement mechanics, and maintaining a safe environment for activity.

The Mann Method PT Exercise program consists of five categories of exercises:

1. Cardiovascular Endurance: sequencing exercises and progressions that enhance cardiovascular endurance over the course of the session.
2. Foundational Exercises: multi-joint movements targeting activation and strength of abdominals, gluteals, hip musculature, trunk musculature, and upper extremity musculature, and improving neuromuscular sequencing.
3. Hip Strengthening Exercises: specific exercises targeting gluteal and lateral hip musculature, transfer patterns, and stability.
4. Visual-Vestibular Exercises: balance and coordination exercises targeting the visual-vestibular systems and integrating stabilization challenges.
5. Stretches: targeted positions and movements addressing muscle tightness, postural asymmetry, postural musculature, and decreased muscle length of gastrocnemius/soleus complex, hamstrings, hip flexors, and lumbar extensors.

The consistent order of the exercises facilitates building a routine, with consistent cueing and a visual chart to support routine and adherence. The cueing focuses on positive feedback and demonstrating how to do a movement, instead of telling the participant what they are doing incorrectly. This accommodates the visual versus verbal learning preferences of adults with Ds and allows for successful experiences and mastery of exercises.

Combining these five groups of exercises, the Mann Method PT Exercise program consists of the exercises described in Table 2.

## 4. Discussion

We used a systems-based physical therapy approach to design an evidence-based exercise program for individuals with Down syndrome (Ds). This resulted in a detailed exercise program addressing Ds-specific impairments in the Body Structure and Function Domain of the ICF model, in addition to targeting strength, endurance, and balance. This development strategy enhances the ability of the exercise program to improve fitness, as well as promote successful motor planning and sequencing of movement patterns, increase functional independence, and improve participation. The mode of delivery explicitly focuses on the learning strengths of adults with Ds, facilitating participant success, confidence, and experience of mastery. Other exercise programs for adults with Ds have been shown to be effective in improving strength, endurance, and balance with Ds [13,14]. However, most of these studies lack information on the content of the programs or the rationale behind the program, nor do they provide information on whether Ds-specific impairments have improved, or how the program affected participation in a wider sense. A recent systematic review and meta-analyses of the benefits of physical therapy interventions in adults with Ds confirmed positive effects on strength and balance [18]. However, in this review, any exercise program was considered ‘therapeutic exercise’ and included in the systematic review, without information whether a physical therapist was involved, or a physical therapy-based approach was used.

This paper offers guidance for clinicians and researchers working with adults with Ds by providing evidence-based recommendations to build or adapt their own exercise program and by providing a detailed description of a program that incorporates all these recommendations. The exercise prescription in this paper is available and usable for all rehabilitation professionals and exercise specialists working with individuals with Ds. Using these recommendations or this program will ensure an evidence-based approach to supporting adults with Ds in their goals and ambitions.

There are many benefits of working with a physical therapist for adults with Ds. A physical therapist can provide support for fitness, work, community activities, rehabilitation, confidence, and body image [17]. However, a few adults with Ds routinely work with a physical therapist. This may be partially caused by the financial burden that emerges when adults age out of pediatric care. There are large differences between states in Medicaid waivers and reimbursement for physical therapy for adults with intellectual disabilities, including Ds. Physical therapists may be utilized less than expected, given the benefits for adults with intellectual disabilities [17]. With increased life expectancy and access to primary and specialized care, such as orthopedic surgery, the need for support from physical therapists has also increased.

Ds is often covered within the pediatric content of standard physical therapy curriculum, with a focus on infants and toddlers. Physical therapists do not receive sufficient training related to medical care and exercise prescriptions for adults with Ds. Individuals with Ds live longer, healthier, and more inclusive lives. This paper provides physical therapists working with adults with Ds in clinical practice with a starting point for evidence-based care.

Research regarding the effectiveness of this program is currently in progress. Future research would benefit from studies including a thorough description of the rationale and content of exercise programs to enable comparisons across programs and enhance our understanding of critical program components for success.

The strengths of this paper include the systems-based approach to designing the exercise program, utilization of the existing literature, and creating a specific evidence-based exercise program that can be replicated in clinical practice in a variety of settings. This has resulted in a thorough evaluation of musculoskeletal and other impairments, and an exercise prescription that addresses those intentionally. However, this does not mean that impairments are the only factors influencing physical activity and exercise in individuals with Ds. Following the ICF model and the bio-psycho-social model, contextual factors and/or social factors also impact health, and we gratefully refer to previous work that has thoroughly summarized those factors, such as Mahy et al. (2010) and Bartlo et al. (2011) [12,75]. The limitations of this study are the non-systematic nature of the literature review, which has inevitably led to missing some published work. However, with the wide range of topics and the need to expand to other populations due to the paucity of papers on certain topics for adults with Ds, performing a systematic literature review was not deemed feasible. The authors used wide search terms, other review papers to find original papers, and the snowball method to minimize the risk of missing critical work.

## 5. Conclusions

Taking a systems-based physical therapy approach in designing an evidence-based exercise program for adults with Ds resulted in specific recommendations for the content and delivery of exercise programs for adults with Ds. We included a detailed description of an exercise program that incorporates unique physiological, neuromuscular, and learning style considerations and maximizes treatment for common movement impairments, while improving activity performance and participation. This evidence-based guidance can empower physical therapists working with adults with Ds in their daily practice, as well as inspire future researchers in providing a detailed description of the design and rationale of their exercise program under investigation.

## Figures and Tables

**Table 2:** Exercises and cueing of the Mann Method PT Exercise program.

	Exercise	Cueing
Foundational Exercises (multi-joint movements that target abdominal activation, gluteal activation, hip stabilization, neuromuscular sequencing)	Squats	15 repetitions up and down – easiest to begin with hand support. 55 cm ball or chair for tactile cue for range of motion. Start standing up with hand support. Feet straight, knees straight. Sit down slowly. Stand up, slow and strong.
	Push-ups	10 repetitions, often easiest to start with push-ups on knees. Start in prone. Hands by chest. Knees bent. Knees together. Push-up—hold 2 s (count 1-2). Slowly down.
	Planks	10–20 s, often easiest to start in quadruped, front plank or high plank. Demonstrate hand position (front plank or high plank). Hands down, knees up. Feet together. Toes pointing down. Eyes up.
	Bridges	10 repetitions, with 5 s hold at the top. Start laying on back. Hands behind head, knees bent. Feet flat on ground, toes forward. Bottom up—hold 5 s (count aloud). Slow and controlled back down.
Hip Strengthening Exercises (specific exercises that target gluteal and lateral hip musculature to improve hip strength and stability)	Hip Abduction	10 repetitions each side with 2 s hold. Start standing next to the wall, one hand on the wall. Best with small target to “kick” for hip abduction. Kick and hold 2 s (count 1-2).
	Quadruped with Reach	5 reps each side with 3 s hold. Start in quadruped. Provide visual and tactile cues for abdominal activation to offset lumbar sway. Reach to visual target (wall or 55 cm fit ball). Hold 3 s (count 3-2-1).
	Seated Marches	15 reps each side. Start sitting in a chair with feet flat on the floor. Hold ball or target in both hands. Ipsilateral—march same side up and down 15 reps in a row. Then the other side. Alternating—hold ball or target at midline and march to target with alternating pattern.
	Standing Marches	15 reps each side. With hand support—start with hands on PT’s shoulders, PT holding ball at midline. Alternating march to midline. Without hand support—start holding ball independently at hip height, march to midline.
	Tall Kneeling Rainbows (PNF D1 Flexion Upper Extremity)	10 reps each side. Start in tall kneeling next to wall holding small sensory ball. Tap ball to floor (lateral trunk flexion), bring ball and arm close to body in scapular retraction and elbow flexion), turn and reach to target on wall 6 inches about head height. Tap, bend, reach. Knees together, feet straight, hips strong, abdominals tight.
	Half Kneeling (Split Stance Surrenders)	5 each side. Start in standing, one hand support on wall or with PT. Step back with right foot (“step and stop”). Right knee bends to floor (“down slowly”). Bring standing knee down to tall kneeling (“together”). Hold with abdominal activation. Bring right foot up (right half kneeling). Stand up. 5 reps right, then 5 reps left.
Visual-Vestibular Exercises (balance and coordination exercises that target the visual-vestibular system and integrate stabilization challenges)	Lateral Tilts	10 times each side. Stand feet hip width. Arms out to sides (90 degrees abduction). Legs straight. Tilt side to side. Shift weight from one foot to the other.
	Rotational Ball Passes/Taps	10 cycles, alternating right and left. Sit/stand by the wall. Hold ball with both hands OR fold hands together. Visual targets at shoulder height. Turn and look. Tap ball to target on wall. Slow and controlled.
	Anterior/Posterior Tilts	10 times right lead. 10 times left lead. Stand in modified tandem stance, with one foot slightly forward of the other. Tilt forward and backward. Keep legs straight. Gaze forward.
	Over-Under Passes/Taps	5 under legs + 5 overhead, alternating over and under. Sit/stand by the wall. Hold ball with both hands OR fold hands together. Visual targets overhead and under legs. Look and reach. Tap ball to target on wall. Start with small movements and work up to larger ones. Slow and controlled.
Cardiovascular Endurance: (sequencing exercises and progressions that enhance cardiovascular endurance over the course of the session)	Sequencing and/or Dynamic Aerobic Exercises	Heart rate >60% of maximal heart rate for at least 20 min of the session. Dance party or other warming-up exercise at the start, foundational exercises that keep heart rate elevated. Effective series for heart rate: Dance warm-up, Squats, Squat jumps, Progressive jumps, Standing marches.
Stretches (targeted positions and movements that address muscle tightness, postural asymmetry, postural musculature, and tight gastrocnemius/soleus complex, hamstrings, hip flexors, and lumbar extensors)	Chest Openers	4 bouts open and close, Standing tall, arms open, chest up, 2 s hold, Lean forward, “hug” to yourself, 2 s hold.
	Overhead Reaches	4 bouts reach up and down, standing tall, arms circle up over head through abduction, 2 s hold, arms down, relax, 2 s hold.
	Single Knee to Chest—Supine	20–30 s hold each side, transfer to floor through half kneel, supine legs extended, single knee to chest, hold 20–30 s, contralateral leg straight with toe up (not in position of hip external rotation).
	Hurdler Stretch—Seated	20–30 s hold each side. Seated on the floor with right leg to the side knee extended, toes up (ankle dorsiflexion). Left knee flexed, left foot against right inner thigh. Right hand to right toes. Left hand on right knee. Hold 20–30 s. Repeat on left side.
	Calf Stretch with Strap	20–30 s hold each side. Supine on the floor. Non-stretchy strap around ball of right foot, preferably wearing shoes. Right hip flexed at 30–40 degrees, right knee extended, right ankle dorsiflexed with support from strap at toes for dorsiflexion stretch. Left hip and knee extended, foot resting on ground. Hold 20–30 s. Repeat on left.

## Data Availability

No new data were created or analyzed in this study. Data sharing is not applicable to this article.

## Supplementary Materials

The following supporting information can be downloaded at: https://www.mdpi.com/article/10.3390/ijerph20043667/s1: S1 Supplementary Exercise Prescription Chart.
