# Supplementary material for: Development of a Physical Therapy-Based Exercise Program for Adults with Down Syndrome
Source: Int J Environ Res Public Health. 2023 Feb 18;20(4):3667. doi: 10.3390/ijerph20043667 (PMC9960831; doi:10.3390/ijerph20043667)
Supplement: Supplementary file 1 [file ijerph-20-03667-s001.zip › ijerph-2125604-supplementary.pdf]

Mann Method PT Exercise Program - Prescription Chart

**Foundational Exercises:** multi-joint movements that target abdominal activation, gluteal activation, hip stabilization, neuromuscular sequencing		
**Exercise**	**Sets and Repetitions**	**Cueing**<br>**(Specific verbal cues and tactile cues for successful movement)**
Squats<br>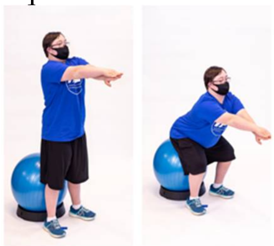	1- 3 sets, depending on progressions<br>15 repetitions up and down	Easiest to begin with hand support.<br>55cm ball or chair for tactile cue for range of motion.<br>Start standing up with hand support.<br>Feet straight, knees straight.<br>Sit down slowly.<br>Stand up, slow and strong.
Push-ups<br>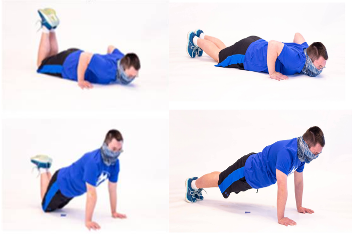	1 set<br>10 repetitions	Often easiest to start with push-ups on knees.<br>Start in prone.<br>Hands by chest.<br>Knees bent.<br>Knees together.<br>Push-up – hold 2 seconds (count 1-2).<br>Control down.
Planks<br>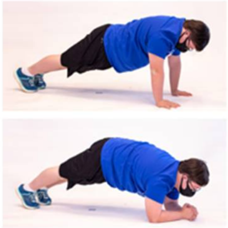	2 sets<br>10-20 seconds each bout	Often easiest to start in quadruped, then move to front plank or high plank.<br>Demonstrate hand position (front plank or high plank).<br>Hands down, knees up.<br>Feet together.<br>Toes pointing down.<br>Eyes up.
Bridges<br>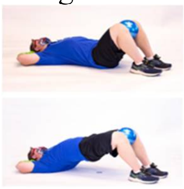	1 set<br>10 repetitions, with 5 second hold at the top	Start laying on your back.<br>Hands behind head, knees bent.<br>Feet flat on ground, toes forward.<br>Bottom up – hold 5 seconds (count aloud).<br>Slow and controlled back down.
**Hip Strengthening Exercises:** specific exercises that target gluteal and lateral hip musculature to improve hip strength and stability		
**Exercise**	**Sets and Repetitions**	**Cueing**
Standing Hip Abduction<br>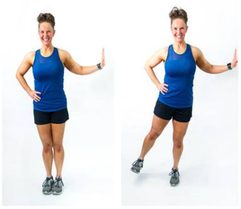	1 set each side<br>10 repetitions each side with 2 second hold	Start standing next to the wall, one hand on the wall.<br>Best with small target to "kick" to for hip abduction.<br>Knee straight, tiny kick to target<br>Kick and hold 2 seconds (count 1-2).

Mann Method PT Exercise Program - Prescription Chart

Quadruped - with reach<br>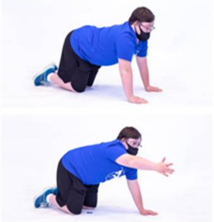	1 set each side<br>5 repetitions each side, with 3 seconds hold each repetition	Start in quadruped.<br>Provide visual and tactile cue for abdominal activation to offset lumbar sway.<br>Reach to visual target (wall or 55cm fitball).<br>Hold 3 seconds (count 3-2-1).
Seated Marches<br>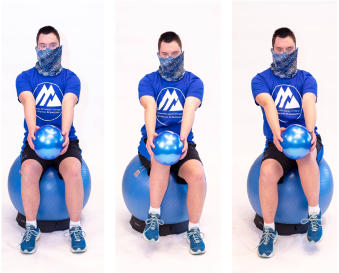	1 set<br>15 repetitions each side	Start sitting in a chair with feet flat on the floor.<br>Hold ball or target in both hands.<br>Ipsilateral – march same side up and down 15 reps in a row. Then the other side.<br>-OR-<br>Alternating – hold ball or target at midline and march to target with alternating pattern.
Standing Marches<br>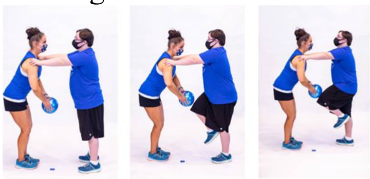	1 set<br>15 repetitions each side	With hand support – start with hands on PT's shoulders, PT holding ball at midline.<br>Alternating march to midline.<br>Without hand support – start holding ball independently at hip height, march to midline.<br>Verbal cues:<br>Bring your knee to the ball.<br>"Tap, tap, tap…"
Tall Kneeling Rainbows (PNF D1 Flexion Upper Extremity)<br>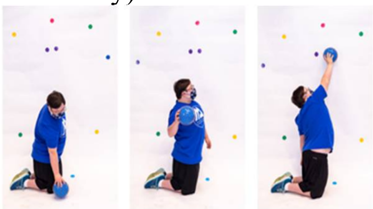	1 set each side<br>10 repetitions each side	Start in tall kneeling next to wall, holding a small sensory ball.<br>Movement pattern: Tap ball to floor (lateral trunk flexion), bring ball and arm close to body (scapular retraction and elbow flexion), turn and reach to target on wall.<br>Visual target placed 6 inches above head height.<br>Verbal cues:<br>"Tap, bend, reach."<br>Knees together, feet straight, hips strong, abdominals tight.
Half Kneeling (Split Stance Surrenders)<br>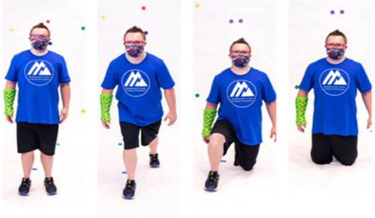	1 set each side<br>5 repetitions each side	Start in standing, with one hand support.<br>Step back with right foot ("step and stop").<br>Right knee bends to floor ("down slowly").<br>Bring standing knee down to tall kneeling ("together").<br>Hold with abdominal activation.<br>Bring "right foot up" (right half kneeling).<br>"Stand up."<br>5 reps on right, then 5 reps on left.

Mann Method PT Exercise Program - Prescription Chart

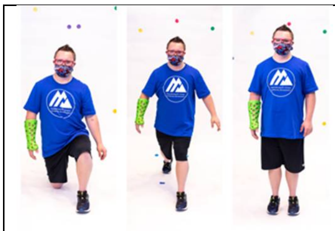		
**Visual-Vestibular Coordination Exercises:** balance and body coordination exercises that target the visual-vestibular systems and integrate stabilization challenges		
**Exercise**	**Sets and Repetitions**	**Cueing**
Lateral Tilts<br>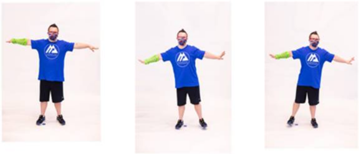	1 set<br>10 repetitions each side, alternating weightshift right and left	Stand feet hip width apart.<br>Arms out to sides (90 degrees abduction).<br>Legs straight.<br>Tilt side to side.<br>Model the tilt and/or provide hand support to start.<br>Shift weight from one foot to the other.<br>Goal: Straight legs, dynamic single leg stance, lateral weight shift, alternating right and left.
Anterior/Posterior Tilts<br>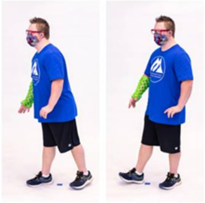	1 set each side<br>10 repetitions right lead<br>10 repetitions left lead	Stand in modified tandem stance, with one foot slightly forward of the other.<br>Shift weight between forward foot and back foot.<br>Keep legs straight.<br>Gaze forward.<br>Provide hand support as needed.
Rotational Ball Passes/Taps<br>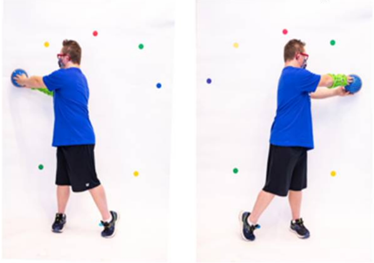	1 set<br>10 cycles, alternating right and left	Sit/stand by the wall.<br>Hold ball with both hands OR fold hands together.<br>Visual targets at shoulder height.<br>Turn and look.<br>Tap ball to target on wall.<br>Slow and controlled.<br>Alternative movement: stand back-to-back with a partner
Over-Under Passes/Taps<br>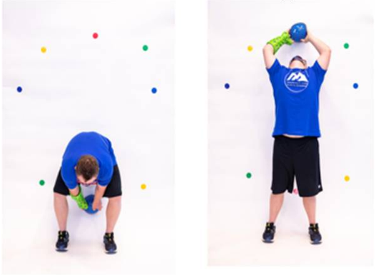	1 set<br>5 cycles<br>Under legs + overhead, alternating over and under	Sit/stand by the wall.<br>Hold ball with both hands OR fold hands together.<br>Visual targets overhead and under legs.<br>Look and reach.<br>Tap ball to target on wall.<br>Start with small movements and work up to larger ones.<br>Slow and controlled.

		Alternative movement: stand with back to a partner and pass the ball from overhead to under legs
**Cardiovascular Endurance:** sequencing exercises and progressions that enhance cardiovascular endurance over the course of the session		
**Exercise**	**Sets and Repetitions**	**Cueing**
Sequencing and/or dynamic aerobic exercises<br>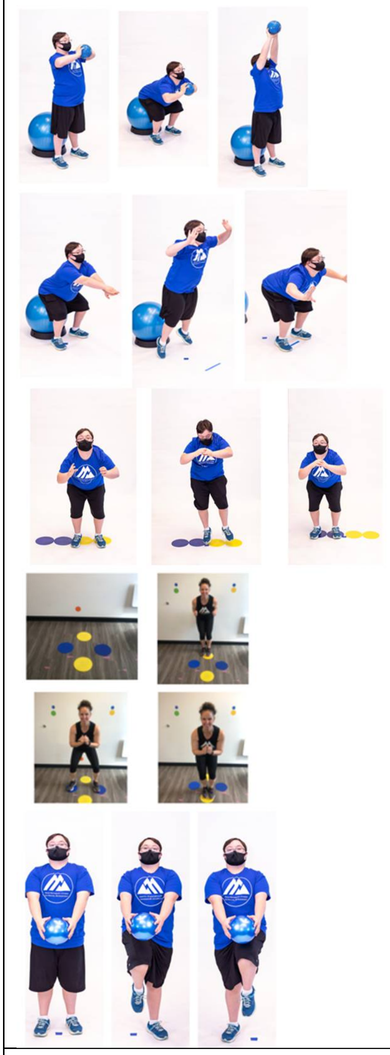	Squat and Reach<br>2 sets of 15 repetitions<br><br>Squat Jumps<br>1 set of 10 repetitions<br><br>Side to Side Jumps<br>1 set of 5 repetitions each side<br><br>Abduction/Adduction jumps: 1 set of 5 repetitions (forward and back)<br><br>Standing Marches<br>1 set of 1 minute-2 minutes	Heart rate >60% of maximal heart rate for at least 20 minutes of the session.<br>Dance party or other warm up exercise at the start, then foundational exercises that keep heart rate elevated.<br>Most effective series for heart rate:<br>Dance warm-up, Squats, Squat Jumps, Progressive Jumps, Standing Marches
**Stretches:** targeted positions and movements that address muscle tightness, postural asymmetry, postural musculature, especially gastrocnemius/soleus complex, hamstrings, hip flexors, and lumbar extensors		
**Exercise**	**Sets and Repetitions**	**Cueing**
Chest Openers	4 bouts open and close	Standing tall, arms open, chest up.<br>2 second hold.<br>Lean forward, “hug” to yourself.

Mann Method PT Exercise Program - Prescription Chart

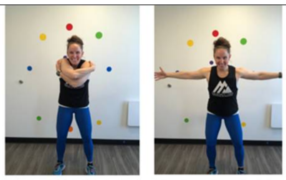	2 second hold with open<br>2 second hold with close	2 second hold.
Overhead Reaches<br>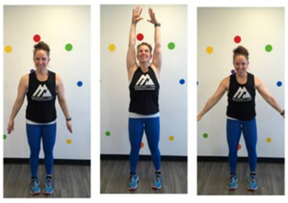	4 bouts reach up and down<br>2 second hold with over<br>2 second hold with down	Standing tall, arms circle up over head through abduction, 2 second hold.<br>Arms down, relax, 2 second hold.
Single Knee to Chest<br>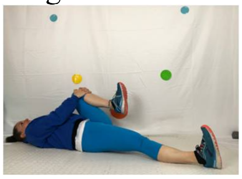	1 set each side<br>20-30 second hold each side	Transfer to floor through half kneel.<br>Lay down in supine.<br>Single knee to chest, hold 20-30 seconds.<br>Contralateral leg straight with toe up (not in position of hip external rotation).
Hurdler Stretch<br>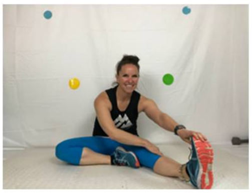	1 set each side<br>20-30 second hold each side	Seated on the floor with right leg to the side, knee extended, toes up (ankle dorsiflexion).<br>Left knee flexed, left foot against right inner thigh.<br>Right hand to right toes.<br>Left hand on right knee.<br>Hold 20-30 seconds.<br>Repeat on left side.
Calf Stretch - with strap<br>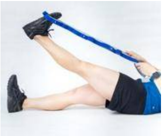	1 set each side<br>20-30 second hold each side	Supine on the floor.<br>Non-elastic strap around ball of right foot, preferably wearing shoes.<br>Right hip flexed at 30 -40 degrees, right knee extended, right ankle dorsiflexed with support from strap at toes for dorsiflexion stretch.<br>Left hip and knee extended, foot resting on ground.<br>Hold 20-30 seconds.<br>Repeat on left.
